# Computer-assisted oblique single-cut rotation osteotomy to reduce a multidirectional tibia deformity: case report

**DOI:** 10.1007/s11751-017-0277-7

**Published:** 2017-03-01

**Authors:** J. G. G. Dobbe, K. J. du Pré, L. Blankevoort, G. J. Streekstra, P. Kloen

**Affiliations:** 1 Medical Imaging Section, Department of Biomedical Engineering and Physics, Academic Medical Center, University of Amsterdam, Room no. L0-113-3, Meibergdreef 9, 1105 AZ Amsterdam, The Netherlands; 2 Department of Orthopedic Surgery, Academic Medical Center, University of Amsterdam, Amsterdam, The Netherlands; 3 Department of Orthopedic Surgery, Orthopaedic Research Center Amsterdam, Academic Medical Center, University of Amsterdam, Amsterdam, The Netherlands

**Keywords:** Additive manufacturing, Malunion, Corrective osteotomy, Computer-assisted surgery, Single-cut osteotomy

## Abstract

The correction of multiplanar deformity is challenging. We describe preoperative 3-D planning and treatment of a complex tibia malunion using an oblique single-cut rotation osteotomy to correct deformity parameters in the sagittal, coronal and transverse plane. At 5 years postoperatively, the patient ambulates without pain with a well-aligned leg.

## Introduction

Long bone malunion can lead to chronic pain, contractures, early osteoarthritis and esthetic complaints [1]. Standard surgical treatment relies on opening or closing wedge osteotomies, rotational osteotomies or combinations thereof. These are conventionally planned using two orthogonal radiographs. A disadvantage of the open-wedge osteotomy is the resulting gap may prolong healing time; if the gap is filled with an avascular bone graft, it may increase the risk of nonunion and reoperation. The closed-wedge osteotomy, on the other hand, produces bone shortening.

With multiplanar malunion, there is malalignment in the coronal, sagittal and transverse planes. Performing the correct osteotomy and the subsequent reduction and fixation is challenging; while angular deformities can be measured using orthogonal radiographs, rotational deformity cannot be measured accurately using 2-D images.

An oblique single-cut rotation osteotomy (OSCRO) [2, 3] can correct malrotations in coronal, sagittal and transverse planes simultaneously by simply rotating the distal bone segment about a line perpendicular to the cutting plane. The advantages of the OSCRO are maintaining a large contact area between the bone fragments and no bone loss. However, the planning and performance of the correct oblique osteotomy is difficult as is the correct rotation angle that realigns the bone fragments.

We report a case of a multiplanar malunion in the tibia. An OSCRO was planned using advanced three-dimensional imaging techniques, and it provided a patient-specific cutting guide for performing a single oblique osteotomy accurately and correcting angular malrotations in the sagittal, coronal and transverse planes.

## Case report

A 32-year-old man (1.85 m, 120 kg) sustained a right lower leg fracture 3 years before presentation which was treated with an external fixator and cast application. His main presenting complaint was lateral pain in his right ankle and, to a lesser extent, in his right knee and back.

**Fig. 1:**
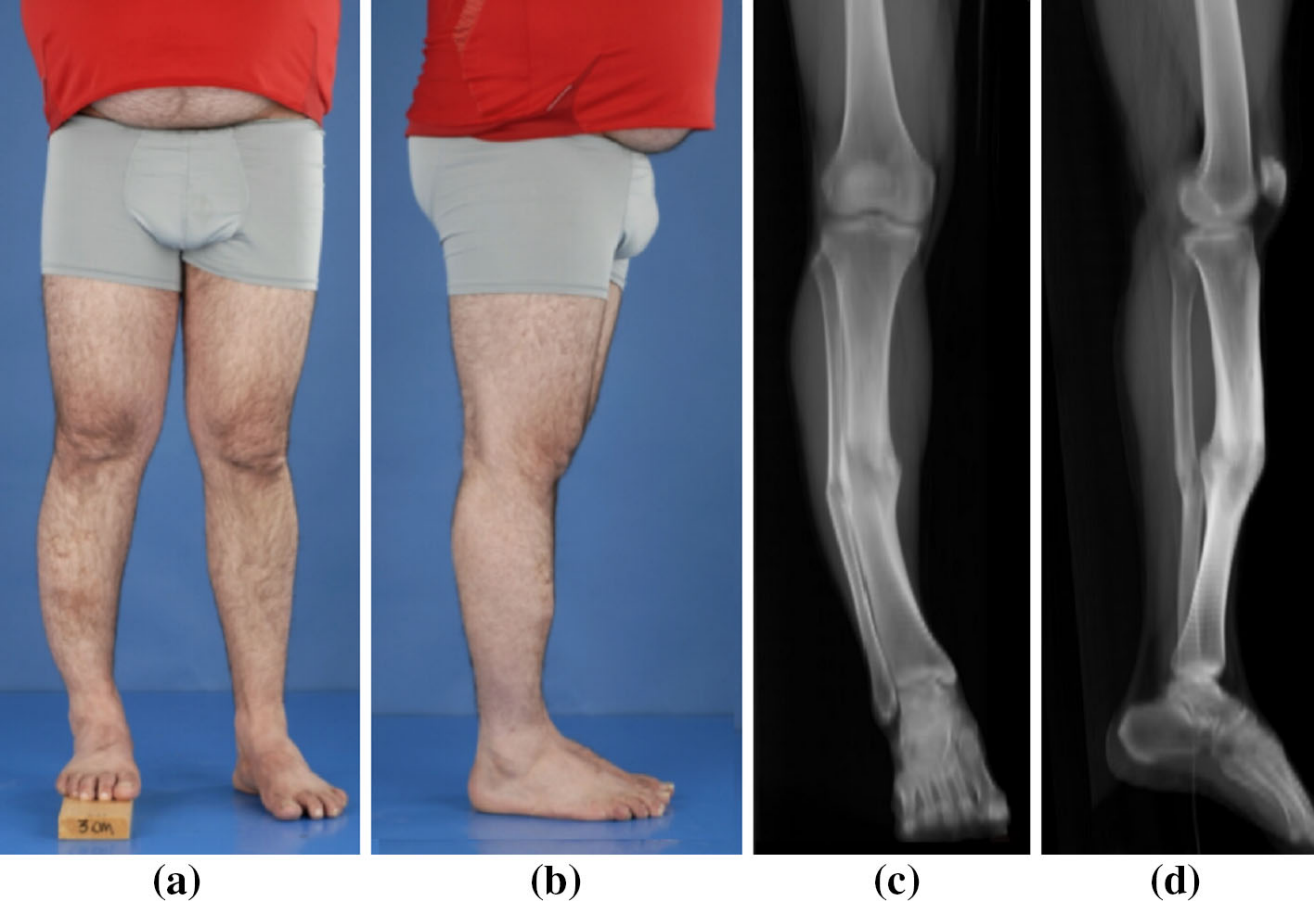
Preoperative malunion deformity of the right lower leg. **a** Frontal and **b** lateral photographic images. Digitally reconstructed radiographic images in **c** AP and **d** lateral view

He presented to our institution with a shorter lower right leg showing a varus and procurvatum deformity (Fig. 1a, b). Clinical examination showed an internal rotation deformity of the distal tibia of approximately 30° which was compensated for by external rotation at the hip. Examination of the right ankle, despite a good ROM, was painful on inversion and tender over the anterior talofibular and fibulocalcaneal ligaments, which was recognized as the origin of his pain. Radiographic examination confirmed a tibia malunion (Fig. 1c, d). He was offered reconstruction of all deformities in one surgical procedure.

Approval of the institutional review board was waived, and informed consent of the patient was obtained.

### Surgical planning

**Fig. 2:**
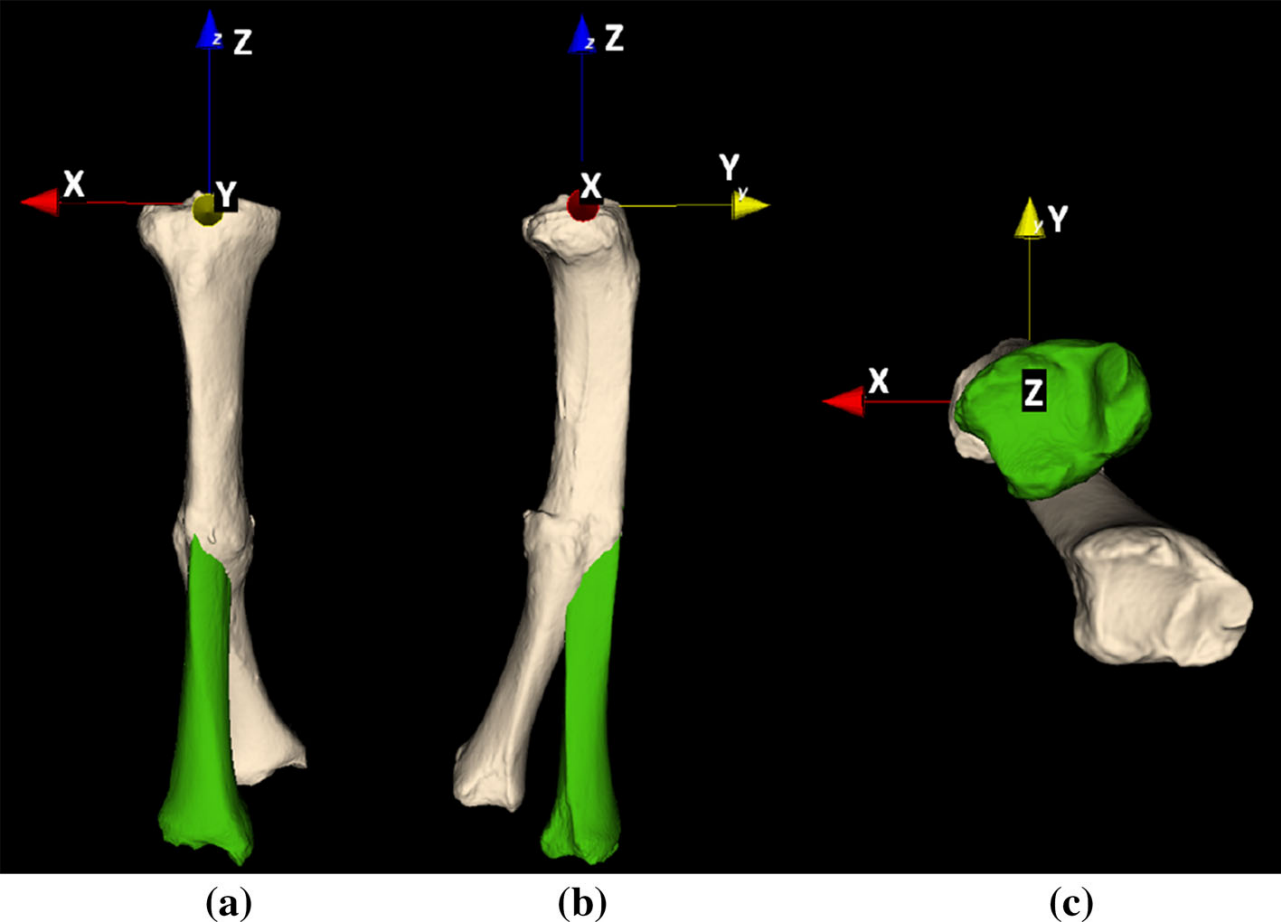
Three-dimensional rendering of affected tibia (*off-white*), proximally aligned with mirrored healthy tibia (*green*) in **a** frontal and **b** lateral view. The affected tibia is clearly deformed in these views but also shows **c** a rotation deformity. The anatomical coordinate system is used to quantify the deformity

A high-resolution computed tomographic (CT) scan at routine clinical radiation dose was made of both tibiae for preoperative planning (Philips Brilliance 64 CT scanner, Cleveland, OH; voxel size 0.45 × 0.45 × 0.45 mm, 120 kV, 150 mAs). The affected right and mirrored healthy left tibia were segmented and proximally aligned to visualize the malunion in 3-D (Fig. 2). Next, a distal and proximal segment was matched virtually with the mirrored image of the contralateral bone [2, 4] to find the correct anatomical alignment. An anatomical coordinate system was defined to quantify the deformity (Table 1). The affected tibia was 25.9 mm shorter and showed procurvatum (flexion) and varus deformation of 6.5° and 21.9°. Internal rotation deformity was 35.9°. These rotations revolve around the three axes of the anatomical coordinate system (*x*, *y* and *z*, respectively).

**Table 1:** Pre- and postoperative malalignment as measured using 3-D image analysis

Malalignment parameter	Preoperative	Postoperative
Sagittal plane angulation	6.5° (flexion)	1.5° (extension)
Coronal plane angulation	21.9° (varus)	3.9° (valgus)
Transverse plane rotation	35.9° (internal)	1.7° (external)
Shortening	25.9 mm	10.4 mm

**Fig. 3:**
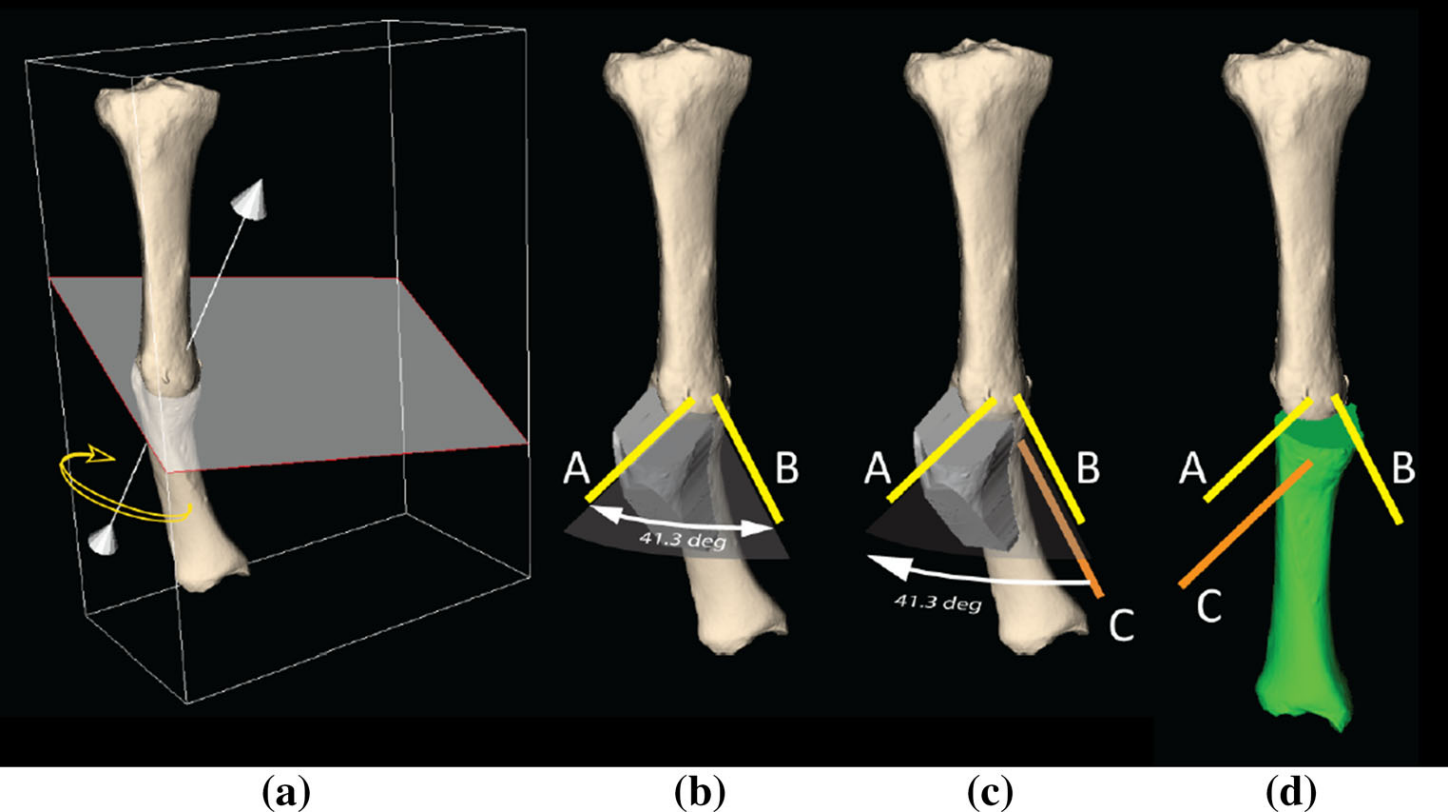
Simulation of surgical treatment. **a** Affected bone and cutting plane showing perpendicular axis of rotation used to bring the distal bone segment in correct anatomical alignment. **b** Affected bone with cutting guide. The *K*-wires, labeled *A*, *B* are inserted in the proximal bone segment and are used to set the angle of rotation. **c** *K*-wire *C* in the distal bone is parallel to *K*-wire *B* before osteotomy, and **d** rotated until parallel to *K*-wire *A* after osteotomy, to achieve rotational alignment

We simulated OSCRO surgery [2, 3] to correct the multiplanar deformity. With this treatment, the bone is cut in an oblique fashion and the distal bone segment is subsequently rotated about the line perpendicular to the cutting plane (Fig. 3a). This single bone rotation reduces the angular deformities in the sagittal, coronal and axial planes. The required rotation angle (41.3°) can be set by inserting two *K*-wires into the proximal bone and parallel to the cutting plane (labeled A, B Fig. 3b). A third *K*-wire (labeled C, Fig. 3c) can be inserted into the distal bone segment and parallel to *K*-wire B. After osteotomy, the distal bone segment can be rotated about the line perpendicular to the cutting plane, until *K*-wire C is parallel to *K*-wire A (Fig. 3d). The OSCRO guarantees optimal bone contact in the cutting plane between the distal and proximal bone segments. To transfer the preoperative planned osteotomy to the patient’s actual bone, a polyamide cutting guide is used (Fig. 3b) which fits the patient’s own bone geometry. Guide production was outsourced (Materialise, Leuven, Belgium). Position planning was performed using custom software [2].

### Surgical procedure

**Fig. 4:**
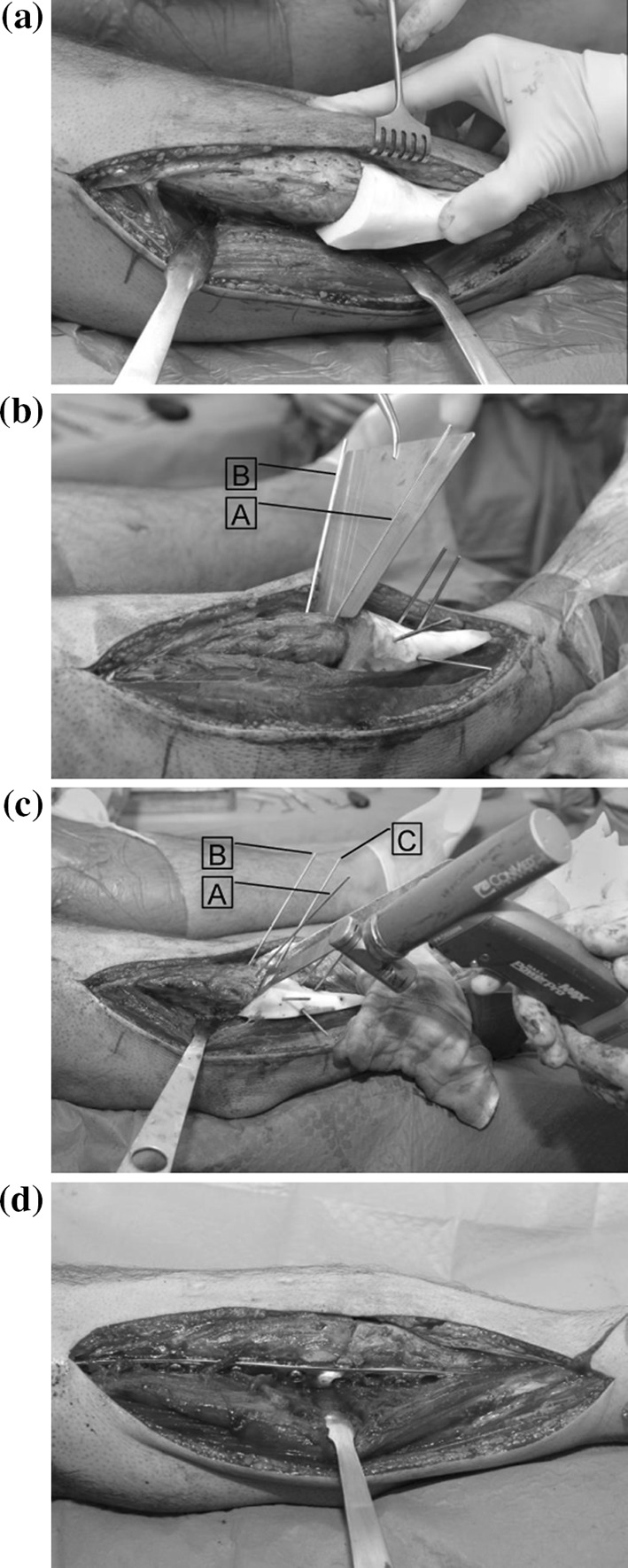
Surgical procedure showing, **a** fixation of the polyamide cutting guide using *K*-wires, **b** Setting the rotation angle between two *K*-wires (*A*, *B*) in the proximal bone using a stainless steel jig. **c** Performing the osteotomy. A third *K*-wire (*C*) in the distal bone segment is inserted parallel to *K*-wire *B*. Reduction is achieved by rotating the distal bone segment until *K*-wire *C* is parallel to *K*-wire *A*. **d** Corrected bone after plate fixation

An anterior approach was used for the tibia with the incision slightly lateral to the tibial crest. After exposure of the tibia, the polyamide cutting guide was applied. It fitted the patient’s bone accurately and was held temporarily using 1.8 mm Kirschner wires (Fig. 4a). Two additional *K*-wires were inserted into the proximal bone and parallel to the cutting plane, to set the angle of bone rotation (labeled A, B Fig. 4b) using a stainless steel angled jig. A third *K*-wire (labeled C, Fig. 4c) was inserted into the distal bone segment, parallel to *K*-wire B. Next, the osteotomy (Fig. 4c) was performed using an oscillating saw under continuous cooling (blade thickness 1.0 mm). An oblique fibular osteotomy was performed to accommodate the tibial reconstruction. This osteotomy was conducted using the cutting guide, under direct vision and continuous cooling. To achieve reduction, the distal tibia was subsequently rotated about the line perpendicular to the cutting plane, until *K*-wire C was parallel to *K*-wire A. Temporary fixation was achieved using additional *K*-wires. Permanent fixation was finally achieved using two 4.5 mm lag screws and a 4.5-mm standard stainless steel compression plate (Synthes BV, Zeist, Holland, Fig. 4d). The plate was contoured to fit bone geometry before fixation with 4.5 mm stainless steel screws. No bone graft was added.

**Fig. 5:**
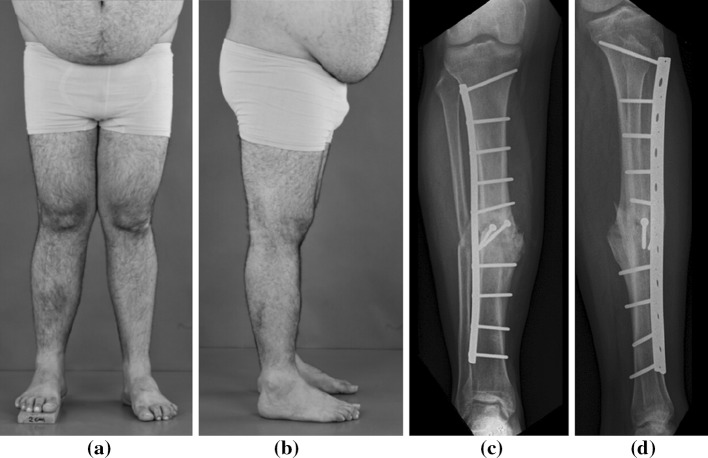
Visualization of surgical result (10 months postoperatively). **a** Frontal and **b** lateral photographic images. **c** AP and **d** lateral radiographs

A splint was applied for 2 weeks to allow for wound healing. The patient was kept toe-touch weight bearing for 6 weeks. Union was confirmed on plain radiographs at 3 months after surgery (Fig. 5). He was weight bearing fully at that time. There were no complications. At review five years after surgery, he walks without a limp, is pain free and has returned to playing soccer.

**Fig. 6:**
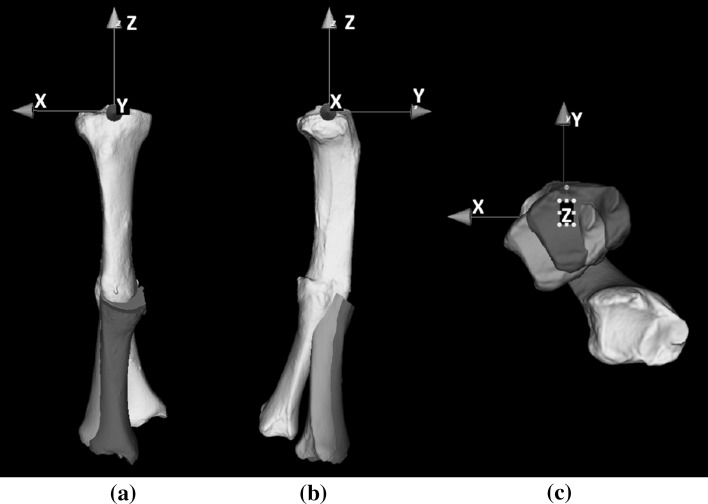
Three-dimensional rendering of affected tibia (*off-white*), distal tibia segment in the planned position (*green*) and distal tibia in achieved position (*red*) showing the residual rotation deformity in **a** frontal, **b** lateral and **c** caudal view

Bone union was also confirmed using a follow-up CT scan (10 months postoperatively). This scan was used for quantitative evaluation of bone alignment. The residual leg length difference was 10.4 mm, compared to 25.9 mm preoperatively. Five of the 10.4 mm was caused by bone loss and was predicted by the software. The remaining 5 mm was due to the surgeon’s choice to have more bone contact between the fragments and less of a step-off pushing into the soft tissues from the sliding of the distal fragment posteriorly (Fig. 6b). Residual displacements in the lateral plane were 10.1 mm in anteroposterior direction and 10.2 mm in mediolateral direction. The residual extension and valgus deformation were 1.5° and 3.9°, and the residual external rotational deformation was 1.7° (Table 1). Figure 5 shows frontal and lateral photographic and radiographic images of the postoperative result. Figure 6 shows a 3-D surface rendering of the affected tibia (off-white), the planned position of the distal segment (green) and the achieved position of the distal segment (red).

## Discussion

Conventional surgical planning is based on anteroposterior and lateral radiographs which provides flexion–extension and varus–valgus deformity angles [5]. Information on rotation deformity in the transverse plane is not available. This renders standard treatment suboptimal for cases in which rotation about the bone axis exists. Preoperative 3-D planning of an OSCRO provides a patient-specific approach, optimal bone contact, and can correct multidirectional deformities. The original length error (25.9 mm) existed due to bone angulation, which was corrected during surgery, and due to bone loss as a result of the fracture and the healing process. The latter cannot be compensated by application of an OSCRO unless a graft is inserted in between the bone segments. The residual translations of the distal bone segment in the transverse plane are considered of lesser clinical importance since they can be compensated for by very small hip joint movements. The residual rotation errors were small (<5°) and are considered acceptable as surgical treatment outcome [1, 6].

In this case, a cutting guide was used to transfer the preoperative planned osteotomy to the patient’s bone. The use of surgical guides allows orienting the cutting blade with high accuracy [7] and without using complex navigation systems. A disadvantage of using guides is the need of sufficient bone exposure for correct placement. In this specific case, adequate bone exposure was created for fixation with a large plate and screws.

When choosing the method of fixation, a surgeon may use an intramedullary nail, which helps with the alignment. However, this may be cumbersome if the intramedullary canal is curved or blocked by sclerotic bone. The nail may disturb the endosteal blood supply further [8] as the periosteal blood supply might be impaired by the exposure and osteotomy. For the motivated and compliant patient, a computer-guided hexapod correction may be an option for this deformity [9]. It provides bone repositioning based on distraction osteogenesis. This external frame is bulky, causing discomfort to the patient and adding the risk of pin track infections; it may not be the correct option for every patient.

Alternative methods for intraoperative bone realignment have been reported. In one method, parallel pin pairs are inserted in the distal and proximal bone segment using a drill guide. After osteotomy, the pin pairs are inserted into a reduction guide to achieve reduction [10]. Other techniques use drilling and cutting guides to predrill holes for subsequent fixation using standard osteosynthesis material [11], or by using patient-specific fixation plates that provide accurate alignment of the bone segments [4, 12, 13]. All these approaches may reduce the surgical error but do not allow any deviation from the preoperative plan in cases where the planned rotation angle appears unfeasible during surgery.

The OSCRO requires a demanding preoperative planning of the oblique osteotomy and calculation of the rotation angle which is not available in general health facilities. This can be considered a disadvantage of the technique. However, the technological aspects of the technique have fully been described [2, 3] and may soon become available from companies specialized in surgical planning. It renders the OSCRO option accessible to and financially viable for most orthopedic surgeons. This enables treatment of complex long bone deformities in most hospitals. The proposed method requires the contralateral limb to be unaffected; this may not be the case for every patient. In this case report, a guide was used to perform the osteotomy while subsequent reduction was achieved by manual rotation of the distal bone segment. Although guided by a simple angled jig, this approach turned out to provide acceptable clinical results.

In conclusion, this case report demonstrates that correction of a complex multidimensional deformity of a long bone is possible using relatively simple means by considering a preoperatively planned oblique single-cut rotation osteotomy.
